# Socioecological factors influencing women’s HIV risk in the United States: qualitative findings from the women’s HIV SeroIncidence study (HPTN 064)

**DOI:** 10.1186/s12889-016-3364-7

**Published:** 2016-08-17

**Authors:** Paula M. Frew, Kimberly Parker, Linda Vo, Danielle Haley, Ann O’Leary, Dazon Dixon Diallo, Carol E. Golin, Irene Kuo, Lydia Soto-Torres, Jing Wang, Adaora A. Adimora, Laura A. Randall, Carlos del Rio, Sally Hodder, the HIV Prevention Trials Network 064 (HTPN) Study Team

**Affiliations:** 1 Department of Medicine, Division of Infectious Diseases, Emory University School of Medicine, 1760 Haygood Road, Suite 300, Atlanta, 30322 GA USA; 2 Department of Behavioral Sciences and Health Education, Emory Rollins School of Public Health, 1518 Clifton Road NE, Atlanta, 30322 GA USA; 3 Hubert Department of Global Health, Emory Rollins School of Public Health, 1518 Clifton Road NE, Atlanta, GA 30329, USA; 4 Emory Center for AIDS Research, Emory University, 1518 Clifton Road NE, Suite 8050, Atlanta, GA 30322 USA; 5 Department of Health Studies, Texas Woman’s University, CFO Bldg – 1007, PO Box 425499, Denton, TX 76204 USA; 6 Division of HIV/AIDS Prevention, Centers for Disease Control and Prevention, 1600 Clifton Road, Atlanta, GA 30333 USA; 7 SisterLove, Inc, 3709 Bakers Ferry Rd, SW, Atlanta, GA 30331 USA; 8 Department of Medicine, UNC School of Medicine, University of North Carolina Chapel Hill, 130 Mason Farm Rd, Chapel Hill, NC 27599 USA; 9 George Washington University Milken Institute School of Public Health, 950 New Hampshire Avenue NW, Suite 500, Washington, DC 20052 USA; 10 National Institute of Allergy and Infectious Diseases, Washington, DC USA; 11 Statistical Center for HIV/AIDS Research and Prevention (SCHARP), Fred Hutchinson Cancer Research Center, Seattle, WA USA; 12 West Virginia University School of Medicine, One Medical Center Drive, HSC-South 2244, Morgantown, WV 26506 USA

**Keywords:** HIV/AIDS, Sexual health, Socioecological model, Women, Minorities, HIV risk reduction

## Abstract

**Background:**

We sought to understand the multilevel syndemic factors that are concurrently contributing to the HIV epidemic among women living in the US. We specifically examined community, network, dyadic, and individual factors to explain HIV vulnerability within a socioecological framework.

**Methods:**

We gathered qualitative data (120 interviews and 31 focus groups) from a subset of women ages 18–44 years (*N* = 2,099) enrolled in the HPTN 064 HIV seroincidence estimation study across 10 US communities. We analyzed data from 4 diverse locations: Atlanta, New York City (the Bronx), Raleigh, and Washington, DC. Data were thematically coded using grounded theory methodology. Intercoder reliability was assessed to evaluate consistency of team-based coding practices.

**Results:**

The following themes were identified at 4 levels including 1) exosystem (community): poverty prevalence, discrimination, gender imbalances, community violence, and housing challenges; 2) mesosystem (network): organizational social support and sexual concurrency; 3) microsystem (dyadic): sex exchange, interpersonal social support, intimate partner violence; and 4) individual: HIV/STI awareness, risk taking, and substance use. A strong theme emerged with over 80 % of responses linked to the fundamental role of financial insecurity underlying risk-taking behavioral pathways.

**Conclusions:**

Multilevel syndemic factors contribute to women’s vulnerability to HIV in the US. Financial insecurity is a predominant theme, suggesting the need for tailored programming for women to reduce HIV risk.

**Trial registration:**

Clinicaltrials.gov, NCT00995176

## Background

Few public health issues in modern times can match the scope and impact of the HIV/AIDS epidemic. In the United States, the number and proportion of HIV/AIDS cases among women has been steady, with women representing an estimated 19 % of all new HIV infections in 2014 [1]. The proportion of Black/African American women with new infections reveals a severe health disparity compared to women of other races [1, 2]. Compared to an overall HIV case rate of 8.0 per 100,000 among females in the US, Black/African American women have a case rate of 41.7 per 100,000, nearly 20 times as high as white females (2.1 per 100,000) and approximately 4.5 times as high as Hispanic/Latino females (9.2 per 100,000) [3]. The HIV epidemic in the US began among MSM. In the earliest days of the US HIV epidemic, the few women who acquired HIV mostly contracted it through injection drug use (IDU) [4]. In subsequent years, the epidemic among US women evolved to one driven primarily by heterosexual transmission. Heterosexual transmission accounted for only 3 % of all female HIV/AIDS cases in 1985 with most of the remaining cases resulting from IDU. Subsequently, heterosexual transmission accounted for a progressively higher proportion of new infections among women, 31 % in 2004 and 87 % in 2014 [1]. Epidemiologic description of the US HIV epidemic clearly documents the evolution of the HIV epidemic from one that occurred predominantly on the coasts among men who have sex with men, through the era of IDU accounting for the majority of new cases among women, to our current state where most women contracting HIV, do so through heterosexual transmission [5].

Community-level factors such as poverty, unemployment, inadequate access to healthcare, the sociocultural environment, and generalized mistrust in the healthcare system may contribute to the racial disparity in HIV/AIDS diagnoses among women [6]. Other drivers of broader health inequities, including lack of employment opportunities, education, housing, social isolation, and perceived political disempowerment and racial or gender discrimination, have also been linked to women’s increased HIV vulnerability [7–12].

These community challenges have been associated with community and social network issues such as greater sexual concurrency among persons living in defined geographic areas resulting from neighborhood gender imbalances [13, 14]. For example, in predominantly Black/African American communities, the impact of disproportionate incarceration of Black/African American men and the associated mortality from community violence creates an imbalanced female-to-male ratio. Such imbalances thereby reduce women’s opportunity to negotiate sexual monogamy in these circumstances [13–16]. Managing such relationship challenges, which are often coupled with the stress of providing for children without financial and social support, has been independently linked to individual-level risk factors [17].

These individual risk factors include coping with antecedent childhood and/or adolescent sexual trauma, substance abuse, depression and mental health disorders, intimate partner violence (IPV), and acquisition of sexually transmitted infections (STIs) [11–13]. Previously established associations such as the SAVA syndemic (substance abuse, IPV, and AIDS) demonstrate the importance of determining intersecting and co-occurring (syndemic) multilevel risk factors to address health issues like HIV in racial/ethnic minority and lower income women in a broader context [18].

Despite the availability of testing and antiretroviral treatment, new HIV infections in the US have been relatively stable over the past decade. The HIV Prevention Trials Network (HPTN) Study 064 assessed HIV incidence amongst women living in communities with high rates of poverty and HIV prevalence, finding an HIV prevalence of 0.32 %, roughly 6 times the HIV incidence estimated nationwide for similarly aged Black/African American women [19]. While previous studies have identified factors that may help explain the vulnerability of women to HIV acquisition, few studies have assessed how these intersecting and reverberating syndemic conditions manifest women’s vulnerability to HIV across multiple diverse US communities [13, 20, 21]. To address the gap, we conducted a substudy of HPTN 064 to examine community (exosystem), network (mesosystem), dyadic (microsystem), and individual factors as they relate to HIV vulnerability within a socioecological framework.

### Theoretical orientation

**Fig. 1:**
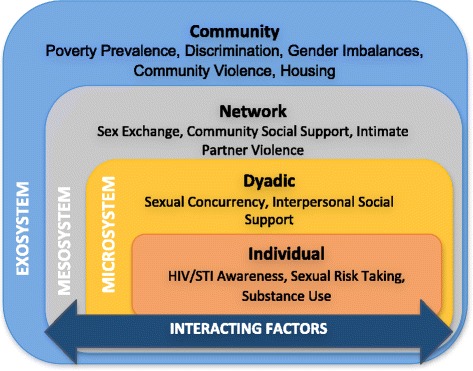
Application of the Bronfrenbrenner ecological model

To examine intersecting factors that help explain women’s vulnerability to HIV, we adopted a socioecological framework to analyze the dynamic interactions across individual, dyadic, network, and community levels in society that place women at risk for HIV acquisition. Our resulting application of the seminal Bronfrenbrenner ecological model (Fig. 1) reflects that individual behavior is influenced and defined by surrounding ecology, environment, and systems [22]. From a conceptual standpoint, HIV risk likely arises from influences at all four of these levels. Our study introduces evidence of the intersections across levels and how these factors operate in a similar manner among women living in discrete geographic areas of the US.

The individual level includes factors affecting HIV risk such as women’s beliefs, feelings, perceptions, and attitudes. Risk-taking behavior and awareness of disease vulnerability are examples. The second level, the microsystem, is theorized to have a strong impact on individual’s health decision-making due to strong interpersonal dynamics. Due to the dyadic nature of personal relationships, an individual often belongs to several microsystems, which intersect based on affiliations among these relationships [5]. The third level, the mesosystem, represents the intersection and linkage an individual has within multiple microsystems (e.g., partnership connections or perceived degree of social support in a network). This is how phenomena such as sexual concurrency may arise when men have multiple female partners [23, 24]. At the next level, the exosystem level, individuals may be affected by indirect forces beyond their direct control. Examples might be forced housing relocation and reassignment, poverty, and racial discrimination. Finally, a macrosystem represents the external system, which influences behavior through policies, laws, and regulations that influence larger cultural and social norms [5].

## Methods

HPTN 064 was a national multisite observational study to determine HIV incidence amongst women living in communities with a high prevalence of HIV infection and high rates of poverty. Details of the HPTN 064 study have been previously reported [19, 25]. As this study sought to understand multilevel and intersectional risks associated with HIV acquisition consistent with the socioecological framework, we used a highly innovative strategy for sampling and recruitment to ensure scientific advancement was achieved as opposed to restating what was known on the topic [26, 27].

First, we identified US metropolitan statistical areas (MSAs) with high HIV prevalence and poverty as these have been documented in the scientific literature as key “hotspot” locations that place women at risk for HIV. Thus, as a fundamental step this multisite study utilized the CDC National HIV Behavioral Surveillance sampling methodology to geographically identify communities from which women at risk for HIV could be drawn. By situating women in the community as a first step, we thus were able to contextualize and describe an array of newly uncovered intersecting relations among “higher and lower-level” sociobehavioral risk pathways [27–32]. As a result, our qualitative sampling methodology was developed to systematically identify women with information-rich narratives that would contribute to scientific advancement that also was fully consistent with best practices for qualitative sampling [26, 33, 34].

Inclusion criteria included women, ages 18–44 years, residing in census tracts or zip codes (New York City) in the top 30th percentile of HIV prevalence and >25 % of inhabitants living in poverty, reporting at least one episode of unprotected sex with a man in the six months before enrollment, and also reporting at least one additional HIV risk behavior (either personal or partner). Using venue-based sampling, eligible women were enrolled between May 2009 and July 2010 from 10 communities in six geographic areas of the US (Atlanta, GA; Baltimore, MD; New York City, NY; Newark, NJ; Raleigh-Durham, NC; Washington, DC). The study was approved by institutional review boards at each site and collaborating institutions, and a certificate of confidentiality was obtained. Routine HIV testing and counseling were performed at baseline and at 6-month intervals, with 6 or 12 months of follow-up. Additionally, participants completed an audio computer-assisted self-interview (ACASI) at all visits that provided for collection of socioeconomic data, alcohol and substance use, mental health symptoms (depression and post-traumatic stress disorder (PTSD)), social support, and sexual behaviors.

Four of the 10 study communities (The Bronx, NY, Washington, DC, Raleigh, NC, and Atlanta, GA) also participated in a qualitative substudy. Each site conducted 30 semi-structured interviews with study participants and 2 to 3 focus groups per age and race/ethnic stratum of women (31 total focus groups of 8–10 women per group). Women were divided according to age group (e.g., 18–25, 26–35, and 36–44 years) and assigned to groups by race and ethnicity as is standard reporting for US government-sponsored studies (i.e., Black/African American or Other race and Hispanic/non-Hispanic ethnicity). We first selected women for interviews based on their alternating participant identification numbers (PTIDs) assigned at enrollment and used the same methodology to identify women eligible for focus groups after the interviews were completed. The interviews and focus groups explored social, structural, and contextual factors that influence women’s HIV risk. Example questions included, “Who do you turn to when you are going through a rough time?”, “What are some things that you believe make a women have a greater chance of getting HIV or an STI?”, and “What can women do to protect themselves from getting HIV/STIs?” Highly sensitive questions about personal experiences (e.g., trauma or victimization) were included in the interview guide only. Focus groups focused on community level factors.

### Ethics, consent, and permissions

We previously reported that at each site and at our collaborating institutions, representative institutional review boards approved the work conducted at their site for this multisite study [35]. Additionally, we obtained a Certificate of Confidentiality from the Office for Human Research Protections in the U.S. Department of Health and Human Services for the entire study, including the qualitative component.

### Sample selection

We used sequential sampling to identify potential interview participants and quota sampling for the focus groups. Focus groups selection was initiated at each site after all interviews had been scheduled.

### Focus group and interview process

Individual interviews were conducted to examine personal risk behavior while focus groups were conducted to examine community perception of risk. We conducted interviews and focus groups in settings that assured adequate privacy and confidentiality, such as the study clinical site, a mobile van, local community-based organizations, or another appropriate public location. Trained interviewers and focus group facilitators were matched to interviewees by ethnicity and gender when possible. We compensated participants for their time and effort. Qualified personnel at each study site recorded and transcribed interviews and focus groups. Each site reviewed transcripts for accuracy and removed geographic and personal identifiers prior to upload into NVivo 8.0 software (QSR International, Australia) for coding [36].

### Transcript coding and analysis

Coding and analysis of study transcripts took place in three steps: 1) Structural Coding, 2) Preliminary Analysis and Member Checking, and 3) Advanced Systematic Analysis.

#### Structural coding and preliminary analysis

All transcripts were structurally coded using NVivo 8.0 and 10.0. Structural coding was intended to identify text associated with a particular question in the interview/focus group guide [37]. Preliminary data analysis took place over two in-person meetings, during which we reviewed and analyzed data for a subset of the structural codes across all sites, described themes, and developed corresponding conceptual frameworks within and across structural codes (e.g., Venn diagrams depicting the intersection of concurrency, financial insecurity, and social support). Community members were involved in all phases of this intensive process.

#### Advanced systematic qualitative analysis

A random subsample of transcripts were selected for advanced systematic coding and analysis [38]. This approach was both compatible with the systematic team-based structural coding already applied to the transcripts and within a grounded theory approach that occurred over the course of a year in iterative cycles [37]. Grounded theory utilizes an iterative, inductive, and deductive process and places great value on simple systematic procedures to allow emergence of theory [39].

During the initial coding phase (Phase 1), analysts reviewed transcripts to develop codes, categories, and to identify emerging themes. We applied open coding to larger segments of text. During axial coding (Phase 2), we noted possible relationships between codes and code groups and developed descriptive subcodes and categories (e.g., childhood violence and adult violence). We then conducted selective coding (Phase 3), which involved reviewing the code categories and discretely coding information relating to that category (hierarchical coding) (e.g., concurrency/personal experiences/feelings). Throughout the constant comparative analysis, analysts restructured codes and refined the codebook accordingly [38]. Phases 1 through 3 were highly iterative and analysts constantly revisited and refined codes until saturation was reached. Saturation was assessed in real time and was defined as the point in the coding process where new codes/themes no longer emerged from transcripts.

### Transcript sub-sample enumeration

We randomly selected 30 % of interview and 51 % of focus group transcripts for advanced coding and analysis due to the large number of transcripts and the resource-intensive nature of Phase 3. These subsample sizes were determined by the achievement of data saturation to establish the coding scheme and thematic content. We enumerated interview transcripts and randomly selected 30 % per site. For the focus groups, we randomly selected one focus group per stratum per site. In addition, we randomly selected one additional focus group from a site that had a large number of focus groups.

### Intercoder Reliability (ICR)

ICR was randomly assessed throughout the coding process for both the interviews and focus groups. ICR rates were calculated using NVivo 8.0 and 10.0 version software programs. We also calculated ICR by hand, using percent agreement and disagreement generated by NVivo. The Cohen’s kappa statistic reflects the extent of agreement among the coders with a range of values from −1.0 to 1.0 (i.e., higher reliability is associated with higher values not likely due to chance) [40]. Thus our k = 0.91 reflects an outstanding level of coding consistency with its value considerably higher than 0.7 which is considered a minimally acceptable level of agreement among coders or raters [40]. For transcripts that resulted in an initial Kappa below 0.80, we asked the two assigned analysts who coded the interviews or focus groups to re-review, reconcile, and revise any coding discordance by revisiting the code definition and reexamining the subject narrative text. When this occurred, we also designated a third independent team member who had not previously coded that particular text to adjudicate any coding discrepancies.

### Statistical analysis

We evaluated differences in characteristics among the broader study population (*N* = 2,099) to those who were subgroup participants in the focus groups and interview components of this study (*n* = 288). Of these, 120 women completed interviews and another group of 168 women participated in the focus group sessions in each location. We examined descriptive statistics, *t* tests, and chi-square tests to determine if there were significant differences between the qualitative sample and the overall study population.

## Results

### Qualitative sample characteristics

The median age of the women in our sample was 27 years, slightly less than that of the overall HPTN 064 cohort (29 years). Most women in our sample were Black/African American (86 %, *n* = 247), also comparable to the total HPTN 064 enrollee population (86 %, *n* = 1802). Additionally, many women reported having a “non-partnered” single status (67 %, *n* = 194) that also was similar to the general study cohort (67 %, *n* = 1410 women). For the 19 characteristics we compared, we found there was no significant difference between the qualitative subgroup and the overall cohort (Table 1). However, the percentage of women reporting weekly drug use among those in the qualitative substudy was lower (15 %, *n* = 42) compared to the broader study population (22 %, *n* = 459, *p* = 0.004).

**Table 1:** Demographics and behavioral characteristics of interview and focus group participants and overall HPTN 064 cohort

Characteristic	Qualitative data (interview or focus group)	Overall data	P
Number of women	288	2099	
Age			
Median	27	29	
25th, 75th % tile	23, 36	23, 38	
Race			0.9685
Non-black	41/288 (14 %)	297/2099 (14 %)	
Black	247/288 (86 %)	1802/2099 (86 %)	
Education			0.4486
Less than High School	100/288 (35 %)	777/2099 (37 %)	
> = High School	188/288 (65 %)	1322/2099 (63 %)	
Marital status			0.1438
Missing	8/288 (3 %)	51/2099 (2 %)	
Married	30/288 (10 %)	159/2099 (8 %)	
Not married, living together	56/288 (19 %)	479/2099 (23 %)	
Non-partnered	194/288 (67 %)	1410/2099 (67 %)	
Household Income			0.4714
$10,000 or less	134/288 (47 %)	933/2099 (44 %)	
$10,001 to $20,000	34/288 (12 %)	225/2099 (11 %)	
$20,001 or more	31/288 (11 %)	197/2099 (9 %)	
Refused to answer/Don’t know	89/288 (31 %)	744/2099 (35 %)	
Number of male partners in last 6 months			
Median	2	2	
25th, 75th % tile	1, 3	1, 3	
Condom used at last vaginal intercourse			0.8562
NA	1/288 (<1 %)	7/2099 (<1 %)	
Yes	55/288 (19 %)	376/2099 (18 %)	
No	230/288 (80 %)	1698/2099 (81 %)	
Don’t know	2/288 (1 %)	18/2099 (1 %)	
Any anal sex in past 6 months			0.7409
Missing	1/288 (<1 %)	5/2099 (<1 %)	
No	175/288 (61 %)	1298/2099 (62 %)	
Yes	112/288 (39 %)	796/2099 (38 %)	
Condom used at last anal intercourse			0.2712
Yes	23/112 (21 %)	143/796 (18 %)	
No	89/112 (79 %)	637/796 (80 %)	
Don’t know	0/112 (0 %)	16/796 (2 %)	
Missing	0/112 (0 %)	0/796 (0 %)	
Exchange sex in past 6 months			0.3703
Missing	2/288 (1 %)	21/2099 (1 %)	
No	187/288 (65 %)	1302/2099 (62 %)	
Yes	99/288 (34 %)	776/2099 (37 %)	
Own Concurrent partnership in past 6 months			0.6938
Missing	1/288 (<1 %)	9/2099 (<1 %)	
No	177/288 (61 %)	1314/2099 (63 %)	
Yes	110/288 (38 %)	776/2099 (37 %)	
Any STI in past 6 months			0.7112
Missing	4/288 (1 %)	33/2099 (2 %)	
No	250/288 (87 %)	1834/2099 (87 %)	
Yes	34/288 (12 %)	232/2099 (11 %)	
At least weekly drug use			0.0040
Missing	1/288 (<1 %)	16/2099 (1 %)	
No	245/288 (85 %)	1624/2099 (77 %)	
Yes	42/288 (15 %)	459/2099 (22 %)	
At least weekly binge drinking			0.9849
Missing	1/288 (<1 %)	32/2099 (2 %)	
No	218/288 (76 %)	1569/2099 (75 %)	
Yes	69/288 (24 %)	498/2099 (24 %)	
Partner risk factor			
HIV seropositive diagnosis			0.5836
No	283/288 (98 %)	2071/2099 (99 %)	
Yes	5/288 (2 %)	28/2099 (1 %)	
Reported STI			0.1270
No	250/288 (87 %)	1884/2099 (90 %)	
Yes	38/288 (13 %)	215/2099 (10 %)	
Illicit drug use			0.0790
No	200/288 (69 %)	1347/2099 (64 %)	
Yes	88/288 (31 %)	752/2099 (36 %)	
Incarceration within previous 5 years			0.2540
No	129/288 (45 %)	866/2099 (41 %)	
Yes	159/288 (55 %)	1233/2099 (59 %)	

### Overview of qualitative findings

As described, coding and analysis took place in two steps, resulting in a total of 1,139 codes for women (10 structural and 1,129 content). We developed total of 3 codebooks and corresponding NVivo databases. As described previously, saturation was assessed in real time, and was achieved in Phase 3 (Advanced Systematic Analysis) of coding and analysis by the time that 90 % of the data were coded. The final ICR was a combined average Kappa score of 0.91 and the ICR for the random sample of interviews was a combined average Kappa score of 0.92.

Our descriptive research yielded themes that described similar linked patterns of HIV risk experience among women. With evidence of multilevel directionality of independent/dependent variable relationships (e.g., poverty driving willingness to remain in a concurrent relationship for partner’s income), variable strength (e.g., thematic magnitude emergent from data), and observed reciprocal factor relationships across levels (e.g., violence and perceived social (non)support), we examined conceptual structures that may align with established behavioral models [41].

We mapped these major themes to the individual, microsystem (dyadic), mesosystem (network), and exosystem (community) levels proposed by Bronfenbrenner’s ecological model. Twelve themes within this rubric consist of (1) Individual-level: lack of HIV/STI awareness and vulnerability, sexual risk-taking, and substance abuse; (2) Microsystem: interpersonal social support, sex exchange, intimate partner violence; (3) Mesosystem: concurrency and organizational support; and (4) Exosystem: poverty, discrimination, gender imbalances, community violence, and housing challenges. An overarching theme emerged on the fundamental role of financial insecurity driving an array of factors contributing to risk-taking behaviors. We present the thematic content according to the Bronfrenbrenner model that reflects effects observed on factors contributing to women’s HIV risk.

### Exosystem: community

A growing body of literature suggests that factors such as poverty, perceived discrimination, community-level gender imbalances, housing instability, and witnessed and experienced crime and violence in geographically defined areas contribute to HIV risk [13–16, 42–47]. Women in our study described these mediating influences and provided insight on how these issues potentially interact, rendering women vulnerable to HIV acquisition.

#### Poverty prevalence

Women explained the toll of financial hardship in their lives and communities and the extent to which individuals will go to procure resources and money for themselves and their children. Many indicated that women often lack enough money or food stamps to obtain food and other household and family necessities for daily living (e.g., diapers, clothing). Such challenges present women with few options to remedy the situation despite having accessible neighborhood food pantries that often place limits on given items to eligible persons and households. A woman summed the desperation experienced by many:

*I know a couple of people around our way that don’t really eat like that or their mother might be on drugs or something and she’ll leave for the whole week and just have them in there. So they got to come over my house and eat dinner or my mother make sure we go take the food over there.*

*- Interviewee, Washington, D.C.*

In addition, participants explained that the level of education in their communities directly corresponds with financial challenges. They reasoned that a lack of formal education results in less marketable knowledge and skill-base to gain employment with decent income potential. Respondents detailed how difficult it is on women who function as heads of households as they are often the (nonvolitional) sole provider to their children. Many are also faced with few options for affordable childcare and have the added challenge of not being recipients of child support from fathers.

*I mean, a lot of people there ain't got no education, period, you know. That's why they ain't got no money, because ain't nobody got no high school diploma or GED trying to make no money.*

- *Focus Group Participant, Raleigh*

*I’m going to keep it real, you know. What I do, I just start making phone calls…Talk to some guys, I’d be like, you know, can I hold something? Oh, what are you gonna give me? [Sighs], and it drives me nuts that I have to do it. To get what my girls need, you know, so. I mean I’m a single mom; I’m by myself. Pops - they don’t want to help, so…*

*- Interviewee, Atlanta*

Many women explained that if they had an opportunity to achieve more education and acquire employment, they would be motivated to work.

*So, yeah it’s hard for when them check and food stamps. That’s why I got myself into little programs and I’m going to program it out too until the country or whoever don’t have no more programs. They pay you $50.00 to go to this program. I’m going for my GED and I’m going for my, I’m going for anything they got so I can get certified. I’m going for food handler license. I’m going for computer, basic computer training.*

*- Focus Group Participant, Washington, D.C.*

A woman in Atlanta summarized that for her, and her children, the attainment of education would inspire hope for a better future.

*There is more to life than the little box you live in. There's a whole other world. There's different things to strive for and you can do anything you want to do…*

*- Focus Group Participant, Atlanta*

#### Perceived discrimination

Among those women who explained that they did perceive distinct differences in HIV vulnerability across communities, they felt this stemmed from fundamental problems like a lack of access to educational and informational opportunities often found in higher income areas of their cities and suburbs. They argued that they often felt left behind and left out of knowing important health information that could help them to protect themselves against HIV. Women perceived that educational disparities (and more direct effects of racial discrimination) did not give certain groups, including Black/African American women, equal opportunity to engage in preventive health behavior, thereby rendering them vulnerable to HIV and many other diseases.

*Yeah. It’s a lot different especially for people who have money and stuff. They’re more educated. They’re more like getting pamphlets at their doorstep, but in the Black community it’s like we have to go out and get the information or people – I mean we have some people who come door-to-door, but at the same time it’s like that information is not easy access for us.*

*- Focus Group Participant, Raleigh*

Another woman described how she dealt with the lack of education in her area for her daughters with personal challenges to protect them from other negative life experiences and to foster their independence.

*And with them going so far out, to different schools that’s not in your area, it’s hard to get to them and be involved in their schoolwork…It’s being there, and making sure they get that education. Letting them know that without that, it’s gonna be harder. All that stuff comes into play. Drugs. Unprotected sex. Pregnancy too young. All that stuff – without that education. Because with that, they know, regardless of what happens in their life, they can take care of themselves. They can be independent – do what they want.*

*- Focus Group Participant, Raleigh*

#### Gender imbalances

Many women described challenges with dating and keeping men as exclusive sexual partners given the considerably low ratio of men to women in the community. Women often attributed this phenomenon to more men in jail or prison, higher male mortality, and having more “undercover men” (i.e., men who have sex with men) available as sexual partners in the community.

*No, I think the most thing that’s getting women these days is sleeping with undercover men and they don’t know it. Because a lot, a high rising of homo-thugs that they’re sleeping with these men, then they’re going back to their girlfriends or their wives. Or they’re sleeping with multiple women to prove to themselves that they’re a man because they feel they’re not one because they’re gay, or whatever the case may be. So I think that’s like the really highest risk of especially black women today.*

*- Focus Group Participant, Bronx*

Men (with a record of incarceration) did not function as a deterrent to sexual relations. In fact, even among women, it is notable that 55 % of the women reported that they were in jail or prison in the past five years (*n* = 159); the broader HPTN 064 study population experienced higher rates (59 %, *n* = 1,233). One woman stated that she observed women becoming less “particular” about their partners’ personal shortcomings. Another focus group participant summarized why she thought there were fewer male partners available to women:

*I’m telling you, if they are not on Rikers, they are gay…And coming here, the first -- when I first came here and having to talk, I just really was like… And I was on the computer doing that little thing, like, “If you are having sex with such and such” -- like, all right: one. But how many people is he having sex with? I don’t know.*

*- Focus Group Participant, Bronx*

Similarly, an interviewee from New York stated:

*There’s so many women available and there’s so many men that’s not available. So many men incarcerated. So many men in mental facilities. So many men at war. They’re [women] limited and they don’t think that they could find something maybe half as good as what they have …So they’d rather settle.*

*- Interviewee, Bronx*

In another focus group, some fear was expressed about women who were knowingly or unknowingly partnered with “undercover men” who could make them vulnerable to HIV and STIs.

*It’s happening a lot more for some reason. But they men really got it over their head and it’s five women to one man so they you know it’s enough to go around. And it’s a lot more undercover brothers out here now that are just leading double lives. So it’s really scary now because I’ve come to the realization that it’s a lot more undercover brothers out here claiming to be thorough and thug and gangster or whatever. And those be the ones so it is it’s very scary.*

-Interviewee, Washington, D.C.

#### Community violence

Community violence is of major concern in these communities. Conversation focused on the role of drugs and gangs, which are a source of income for the communities, as major contributors to the issues of crime and violence in the neighborhoods.

*Mmm mmm, alcohol use and you know street walking and everything, shooting, gun shots, people beating each other up, people hollering, jumping fences and shooting cats with the BB gun and just everything. It’s just everything. They just want to kill everything that’s walking. So, it’s just a very like I said destructive, depressing neighborhood.*

*- Focus Group Participant, Washington, D.C.*

Many women indicated that they had a hard time coping with the extent and magnitude of violence in their neighborhoods.

*… I mean it’s so bad that they shoot guns that I feel like they coming through my living room window. I be telling my kids get down, they get in the middle of the floor, they’ve been getting in the floor. So, everybody just get down on the floor. So, it’s just terrible. I mean I don’t feel safe at night when I sleep because where my bedroom window is it feel like somebody could just come through there shooting or my house been burglarized already. They stole some things out of my house that, so it’s terrible around there.*

- Focus Group Participant, Washington, D.C.

In an effort to protect themselves and their children, some stated that they would like to leave the neighborhood to avoid becoming crime victims, yet many were financially unable to move. Their immobility often transpired into self-isolation as a key coping mechanism.

*So we’ve got -- we’ve got each project against each other. So you’ve got the shooting, you’ve got the fighting, you’ve got the gangs. And, you know, so it’s too much. I have a two-year old, you know, so it’s not too much that I want him to see.*

*- Focus Group Participant, Bronx*

#### Housing challenges

Women in our study described additional structural barriers such as negative housing circumstances (e.g., mold, insect infestation) associated with their physical dwellings that resulted in unsanitary and unsafe conditions. Yet, many women offered narratives on their limited mobility due to financial circumstances and restrictive housing policies. A woman from Washington, D.C. described her challenges getting an apartment and and retaining a place to live despite the health hazards it presented:

*Because of my credit I was trying to find an apartment cause I was staying with somebody when my mother lost her house. So I was staying with somebody so I was like putting in applications. And someone stole my identity so it was hard for me to get an apartment. So my girlfriend she had bad credit so it was like word of mouth and I got it like that so. Yeah they lived there they just moved there like a week before I did. Well a month before I did. And she told me about it so. And I went and it worked out. I stayed out there for a year and a half. I liked it though but my apartment was, the building was hazardous, getting hazardous, mildew, mold and all of that so…*

- Interviewee, Washington, D.C.

Others stated they were forced to remain in apartments or dwellings assigned to them through housing programs because they had no other options due to limited financial resources. Nearly all described the mental and physical effects housing challenges had on themselves, their children and family members, as well as those living in their buildings, apartment complexes, and neighborhoods. One woman summarized what it is like to live in her building and in her apartment, both of which are structurally deteriorating, and the extent of sexual risk behavior and drug use occurring in such an environment:

*People die, the drugs, the apartment building, the whole building breaking down. If it’s not something in your house tearing up, it’s something in the building or somebody been caught having sex in the steps. Somebody OD’d [over-dosed] on the elevator.*

- *Focus Group Participant, Washington, D.C.*

### Mesosystem: network

Individuals are connected to and operate in many social systems and networks that constitute the “mesosystem,” a level representing intersections across contexts. At this level, we identified two major themes including how sexual concurrency and (lack of) organizational support function to promote women’s HIV vulnerability.

#### Sexual concurrency

Our quantitative data revealed that a substantial proportion of our study sample reported being a partner to a man with multiple relationships (38 %, *n* = 110). The qualitative data offered many perspectives on the subject of sexual concurrency. Within this domain, we learned that women tolerate their male partners’ sexual concurrency with other women as involvement with that partner privileges their relationship. By having an elevated status as “main partner” to her man, women indicated that they felt validated. Often she knew that other women were simultaneously competing for his interest. One woman summarized the situation for women in “committed” relationships who knew of their partner’s “external” sexual relations:

*Or they look at it like, “I’m wifey, he is getting what he wants, and I’m going to be his number one. No matter what, he's going to come home to me tonight.”*

*- Focus Group Participant, Bronx*

Elsewhere, a woman commented that other women may experience a boost of self-esteem when they feel that they are elevated socially, sexually, and emotionally by the way a man treats her even if she knows or suspects he has other female partners:

*I guess ‘cause they feel like they’re getting treated like a queen at the time and nobody else can probably do or say - - I don't know, I guess, I mean I really don’t know. I mean maybe I guess because they’re not getting treated the way they want to get treated with other people. Maybe he’s just saying and doing stuff that’s making her feel good.*

- *Interviewee, Raleigh*

Similarly, participants explained that most people, especially women, remained in concurrent relationships with men because they did not want to suffer from loneliness. She stated:

*A lot of women can’t be by themselves. They have to be with a man because they don’t want to be alone. How is that? If you really -- when you go through a problem and you let him go, you can work on yourself. You can try to do things better for yourself, but to say, I can’t live without him or I can’t live without a man knowing that he did this is, no no.*

*- Focus Group Participant, Bronx*

For some women, financial reasons factor into their willingness to stay with a male sexual partner who they know to have other female sexual partners. In Atlanta, one woman summarized what she perceived to be the reasons many women tolerate his relationships with other women:

*I think it’s that comfort and security that they have with that person. Okay well, he’s paying my bills I’ll just deal with this. I’m going to tolerate what he’s done, if it’s right or wrong…*

*- Focus Group Participant, Atlanta*

Overall, many women believed that people, both women and men in their communities, engage in high frequency of overlapping sexual partnerships. They described that women are often aware or have strong intuition of their partners’ indiscretions and they have direct knowledge of their friends’ or the friends’ partners’ concurrent sexual behaviors. Yet, it is important to note that only a few women reported concurrency or suspected concurrency in their own relationships. A woman commented on women’s tendency to emotionally and socially protect herself when making social comparisons:

*I think women know when a man is cheating on them. They just don’t want to admit it. They don't want to know ‘cause they don’t want to be embarrassed around their friends that they’re fronting for and they make them think they’ve got the perfect relationships.*

*- Interviewee, Raleigh*

#### Organizational support

We observed important distinctions on actual and perceived support at the community level. Women explained that instrumental support such as food, shelter, clothing, and healthcare services were available in their communities. They felt that such services were necessary for their survival, especially as many indicated that family, friends, and neighbors could not be trusted to offer any instrumental or emotional support. Thus, the women described turning to caseworkers, community-based organizations, rescue missions, and women’s health clinic staff for any type of support. As one woman from Raleigh stated, she turned to community-based organization staff, “…cause I feel like they can help you, answer your questions and stuff.” Another woman from the Bronx explained the critical role of formal organizations to be leaders on developing needed support groups for women in the neighborhood. Such opportunities provide women with emotional support, social connection, and access to information and coping strategies that help them to protect their health and well-being:

*I have a support group, you know, I have a support group, I go to therapy. You know, because when you’re raised in neighborhoods like this, you know, and you’ve been here all your life, you know, you’re subjected to a lot of things. You know? You’re subjected to abuse, you’re subjected to violence, subjected to domestic violence, you know?*

*- Focus Group Participant, Bronx*

Importantly, local and neighborhood-based organizations hold trust with these women. They connect with the staff and volunteers of the organizations (e.g., community-based organization case works, peer health navigators) and form trusting relationships with the individuals who can help them work through difficult problems such as staying in recovery, addressing homelessness, and other critical needs.

Finally, women described the importance of having other female volunteers deliver educational support to children in the community. An Atlanta interviewee explained the importance of having homework and childcare support present in her apartment complex recreational facility as it addresses vital needs of many women for their children, especially as men are not around and able to assist with after-school activities.

*Okay where I stay at, they have a breakout center. They keep your kids from 2:30 afternoon to 6 o’clock in the afternoon. They help the kids with their homework and everything so I mean, it’s women doing that. So I think that’s one way they helping the children out in the community because they…I mean it’s a program, a free program…A lot of people need that sometimes. Free!*

*- Interviewee, Atlanta*

### Microsystem: dyadic

The effects observed at this level included three major factors directly affecting women’s vulnerability: sex exchange in the community, varying peer and familial social support, and intimate partner violence.

#### Sex exchange

To survive, many participants reported that they and/or people in their communities turn to any means necessary to obtain money or food for themselves or their families. They may turn to selling sex or exchange their bodies for drugs, housing, food, or other living needs. During a focus group, one woman stated:

*There's girls out here that I know that you would never imagine in your wildest dreams that they would do the type of things they do. But crunch times, you got your kids, you got to feed your baby. You got to feed yourself. They out here streaking or whatever you want to call it just to make sure that they good and they baby good, because at the end of the day, if they ain't watching out for their kids, ain't nobody else gone do it. I mean, people is resorting to any and everything at the end of the day, just to make sure they're getting – they're gonna eat, and their baby's gonna eat.*

*- Focus Group Participant, Raleigh*

#### Social support

Women reported receiving some personal social support from their mothers or friends. Yet, the majority of women stated they felt a general lack of instrumental (or physical “resource-based”) and emotional support. Instead, they perceived more negative support from family, friends, and peers (e.g., disapproval of life choices). Many of the women reported having very few people they could turn to in times of need (some referred to having only one person) they could claim in their support system (i.e., mother or friend). Those who reported little or no emotional or instrumental support (e.g., childcare for employability) also explained that too often the women in their communities are simply disingenuous and have hidden motivations if they extend offers to help. An example of this type of behavior is described in a quote by a woman who described what happened after she confided in a “friend.” According to this woman, the acquaintance betrayed her confidence to gain material support (or money) from a man with whom she shared the story:

*So she goes back and tells him all my business, because she might need a three-piece - $1.99 for Church’s or some money or some toilet paper…So that’s why I say I really don’t turn to nobody no more. But I keep her real close to me. Because if something happens to me, she gonna get hurt first.*

*- Interviewee, Raleigh*

Women stated that they were often distrustful of family members, friends, and neighbors, especially those who previously were or currently are critical of their lifestyle or life circumstances. Women therefore self-isolate in response to negative social support and withdraw from social circles, often at some hidden cost. This resulted in distrust of other women and men. Reasons why women would not want to confide in others was summarized by an interviewee:

*So you can’t tell everything to anyone, because you don’t know who they’re going to tell or if they’re going to tell this person and that person’s going to tell another person.*

*- Interviewee, Bronx*

#### Intimate Partner Violence (IPV)

The women in our study chronicled a considerable amount of traumatic experiences associated with domestic violence and IPV. Detailed accounts emerged on past and current physical and emotional partner abuse episodes, both witnessed within families and personally experienced. As victims and household bystanders to partner violence, women echoed how this type of violence fostered their strong distrust of men. In Raleigh a woman described her history:

*Well, growing up, my mother used to get beat up on a regular - um I been around my mother getting her tail beat most of my life, all the way up till I was 18 when I left home. Then I'd be around my sister, the one up there, she’s just like - her boyfriend all the time. Then I got into a bad relationship where it won’t like he was hitting on me. He just was yelling and screaming all the time, playing with guns, putting guns out and stuff like that. But pretty much all the time.*

*- Interviewee, Raleigh*

Another woman from Washington, D.C. described her experience with a jealous husband of whom she suffered his abuse for some time that became a norm in their household:

*My husband used to beat me and I did not you know me living up in D.C. I wasn’t raised like that men beating on you or nothing. My husband was jealous…*

*- Focus Group Participant, Washington, D.C.*

Typically, women detailed that male partners exhibiting violent behavior against them and others ended up incarcerated for some time, thereby ending their relationships. An Atlanta woman stated:

*He’s been incarcerated now for like, 3 years now and I felt unsafe cause he use to abuse me…I don’t know why he started abusing me and stuff.*

*- Interviewee, Atlanta*

Those who survived violent acts perpetrated by male partners described other ways out of these relationships. Many stated that men took advantage of their low self-esteem and exploited their vulnerabilities. Yet, they described their resiliency and process of transformation to stand up to the abuses experienced after some catalytic event such as going to jail or prison, losing their children, or some other major life change. A woman in Washington, D.C. spoke of her attitude shift toward her partner and her desire to move past her history of abuse:

*…can’t nobody touch me. And if you do, there’s consequences. That’s where I was. I went from there to there in 1996. From 1996 until now, I’m here. My tolerance - everything - process of elimination. I went through that whole process myself. I have to pinch myself. I have to get my mind right. I have to get my body right. I have to get my head right. Spiritually, emotionally, everything. I have to do all of that myself…*

*- Interviewee, Washington, D.C.*

### Individual level

Three major themes emerged on the interdependent role of HIV/STI awareness, risk-taking behaviors, and substance use that linked to themes observed at the individual/dyadic (microsystem), network (mesosystem), and community (exosystem) levels.

#### HIV/STI awareness

The majority of participants reported that they have thought about their own vulnerability to HIV and/or STIs in relation to those they observed among other women in the community. Although the median age of women participating in the qualitative substudy was 27 years of age, women estimated that they were at low personal risk of acquiring HIV because of the older age of their male sexual partners. A woman in Atlanta explained:

*As of being younger, everybody just having sex with anybody and just doing it and now I just think the guys I…I think the guys I’m dealing with, they old…they got a older mind frame and they not into popping this female, jumping this female, go to the club pick up this female.*

*- Interviewee, Atlanta*

Despite their lack of perceived vulnerability to HIV, a majority of participants reported that they knew someone with HIV or STIs. In the Bronx, a woman spoke of general risk denial among women she knew. She simply stated that most women “…they don’t think it could happen to them.”

#### Sexual risk taking behaviors

Most participants reported the lack of condom use, having multiple previous and/or current sexual partners, and having a lack of knowledge of partners’ history as major risk factors for HIV/AIDS. Many participants explained that they do not use condoms with partners whom they consider to be in a steady or long-term relationship.

*Yeah, I mean we have them in the house. But it’s not like I'm going to go and grab one, you know, every time we get ready. ‘Cause I guess ‘cause I’ve been around him for so long, it’s just it doesn’t even come up in our conversations… I guess ‘cause I’ve been around him for so many years, you know. I guess I just fell into that comfortable mode as far as not using protection.*

*- Interviewee, Washington, D.C.*

Although the majority of women indicated that they were not in a “non-partnered” relationship (67 %, *n* = 194), a woman from the Bronx described that relationship duration is a criterion for determining if condoms are used.

*I'm going to be honest with you. If I'm with this man for ten years, I'm not going to be using a condom, if I'm with this one man for ten years. If I'm with this man for one year, yeah, we going to be using condoms.*

*- Interviewee, Bronx*

Another facet of risk-taking behavior involved the association of substance use to condom decision-making. They offered many accounts of women they knew who engaged in more risky sexual behaviors under the influence of drugs and alcohol.

*It might, you might be in the heat of the moment, might be on some alcohol anything, like boom! You know it might not happen. You might think, you might not think about it, oh darn, get a condom. You know, you could be in the heat.*

*- Focus Group Participant, Atlanta*

#### Substance use

Many women described a personal history of substance use, including crack, alcohol, marijuana, ecstasy, and other hallucinogens that they used to cope with other life factors such as a lack of social support, partner violence, and other negative life circumstances. In Washington, D.C., one woman summarized why she began using:

*Okay well it started in [name of state] you know what I mean cause I kept saying oh I need my, I used to say it was self-medicating. Cause I was going through like a lot and then it was like I got depressed real bad and my son went to live with his father. So I just my attitudes like, I just couldn’t cope. So it’s just when I got high I didn’t think about nothing. I didn’t care pretty much I used to say it was self-medicate. But then it was like when I removed myself from the domestic violence part of it, it was like I just started seeing a different outlook.*

*- Interviewee, Washington, D.C.*

Women reported that their consistent use of substances rendered them vulnerable to HIV, having impaired their judgment especially with respect to sexual partners. One woman stated that she did not even think about using condoms with male partners while she binged on alcohol:

*Oh I came from the club that’s what happened I came from the club and probably was drunk. That’s how I got my first daughter. I was drunk and it was too late, too late.*

*- Interviewee, Raleigh*

It is important to note that the quantitative data indicated that, on a weekly basis, women in our sample consumed fewer drugs (15 %, *n* = 42, *p* = 0.004) and were less likely to engage in binge drinking (24 %, *n* = 69) compared to the larger study sample’s drug (22 %, *N* = 2,099) and alcohol use (24 %, *n* = 498). This suggests that women were cognizant of the role of substance abuse on their risk of acquiring HIV or STIs. Several described their substance abuse experiences in the past tense, indicating personal journeys with recovery. By reducing or eliminating use of substances, they recognized that they could avoid acquiring HIV. One woman spoke of her personal struggle with crack addiction and that of her male partner who was living with HIV. This raised concern for her about her vulnerability to HIV as they shared drugs, of which their paraphernalia was also shared with his wider social network. She explained how using drugs placed her directly at risk of contracting HIV from her partner whom she remained with for over 10 years. With treatment for crack addition, she was able to liberate herself from a challenging relationship with a man living with HIV.

*Because he’s sick. He has the virus so…Yeah everybody was on crack, everybody was on crack. Except for the wheelchair; he was a weed head. ..It was all about getting high.’Cause today I’m working and I can wait for the next paycheck to get what I need. I don’t have to go out because I’m not using anymore not under the influence. So I’m thinking more clearly now.*

*- Interviewee, Raleigh*

## Discussion

Findings from HPTN 064 qualitative data from 288 women selected from the HPTN 064 study provide insight into the factors driving women’s risk in the US on a large scale, as this is among the first national multisite, multilevel analyses that qualitatively explores nested and interacting factors contributing to women’s HIV risk in the US at multiple diverse sites. The findings correspond with quantitative studies that examine the role of poverty, such as homelessness, disintegration of neighborhoods, housing challenges, and overall lack of social capital in geographically defined areas, and how these factors may facilitate HIV transmission [48–53].

Our findings support the fundamental role that financial insecurity plays in HIV risk among women in certain communities in the US [54–56]. Participants indicated that their reported high-risk behaviors, including unprotected anal or vaginal intercourse with concurrent partners, stemmed from challenges related to acquiring food, housing, and other necessary provisions for themselves and their children.

This study offers further evidence of important structural considerations at the community (exosystem) level [9, 57]. Women described conditions of poorly managed, unstable housing conditions and unsafe neighborhoods characterized by violence and substance abuse issues, all previously correlated with HIV risk behavior [45, 58–60]. They describe living in these environments as chaotic and stressful, with many women unable to escape their neighborhoods due to financial constraints. Economic immobility experienced by women offers little or no choice in housing and physical environment. This aspect alone contributes to the psychosocial stressors they experience on a daily basis [61]. Consequently, personal or witnessed HIV/STI risk-taking behaviors are described as being induced by increased stress and anxiety associated with unsafe and unstable housing environments.

Additionally, there was consensus that the prevalence of substance abuse in neighborhoods contributed to risk-taking behavior and fueled unnecessary crime and violence. Sex exchange was also described both in terms of personal means to obtain financial and in-kind resources. Trading sex for money, housing, food, drugs, or gifts is a manifestation of coping with living in an area characterized by high unemployment, incarceration, and poverty [62]. Thus, as we and others have identified, overall financial difficulties and poor living conditions exacerbate overall stress endured by women that poses significant barriers for HIV risk reduction [63].

At the individual/dyadic (microsystem) and network (mesosystem) levels, women described key issues that reciprocally interact with other upper- and lower-level socioecological factors, including a prevalence of community-wide concurrency and IPV as well as a lack of perceived social support and, relatedly, self-imposed alienation from others stemming from distrust [64]. Yet, important information emerged on the vital role of support from community agencies. Women expressed a moderately high level of trust in the staff and volunteers of these organizations who lent tangible support to them as well as emotional comfort in times of distress and need. Thus, leveraging the role of community-based organizations will facilitate the development of effective multilevel interventions to reduce HIV risk among women living in these communities [65–68].

With a substantial number of women reporting being a partner to a man with multiple relationships, women described it as a more widespread phenomenon constituting a community norm [23, 69]. This norm is driven by broader societal/community-level (exosystem) issues such as violence and criminal laws associated with substance abuse, resulting in greater incarceration rates compared to other neighborhoods and thereby constraining sexual partner options [24, 70–72]. They explained that they are very aware of the role of men’s sexual concurrency with multiple women in the community on HIV risk. They reported this from direct observation of men and women entering and leaving residences for sexual activity, having conversations with others about “stepping out,” and experiencing or knowing people living with HIV and STIs [70].

Sexual concurrency, also linked to experiences with IPV, is also associated with women’s HIV risk [73]. Women detailed their personal experiences with physical and mental abuse inflicted by partners, often explaining IPV as a multigenerational cultural norm witnessed or experienced by mothers, grandmothers, sisters, and female cousins who may not necessarily offer social support to them [74]. IPV traumatic experiences with male partners often arose through confrontations about his “stepping out” on her or about sexual liaisons with other women.

Similar to other studies that report on women who experience PTSD following IPV [73, 75], our participants also signaled their inability to effectively address concurrency or walk away from partners who offer financial support [76]. In these circumstances, financial dependency reduces women’s independence and her power to advocate for herself and her children’s health and well-being. Thus, at these levels, trust and dependency issues emerge as significant challenges. Women see themselves as being “in debt” to others. These factors directly restrict women’s ability to confront partners for fear of violent retaliation, to negotiate condom use, or to identify those she can trust to identify instrumental resources to navigate away from these risk circumstances [77, 78]. Given the importance of these issues, integrated HIV-related interventions featuring partner communication and financial empowerment may offer great promise for addressing these challenges.

Finally, at the individual-level women described substance use and sexual risk-taking despite having observed a high prevalence of those living with HIV in the community. Women described the prevalent use of drugs and alcohol by members of their community as a coping mechanism to their socioenvironmental challenges including sexual partner norms (i.e., concurrency), IPV, and living environment violence [79, 80]. Thus, although the linkage between substance abuse and HIV risk taking behavior is well known at the individual level [81], our qualitative analyses identified that these community challenges influence their willingness to engage in social relationships with others in their community for fear of violence, harmful gossip, and/or some other form of retribution inflicted on themselves, their children, or people close to them. Often choosing to withdraw from social interactions with others beyond one or two persons (usually a child or other family member), women describe that they have turned to sex with men who may have behavioral risk characteristics (e.g., living with HIV) and/or use substances, thereby altering their own risk for HIV acquisition in spite of having a relatively high HIV/STI awareness and knowledge of how HIV is transmitted [82, 83].

Our findings therefore corroborate those that link more distal factors such as disadvantaged neighborhoods to proximate health behaviors and outcomes including negative drinking norms, sexual risk taking, stress reactivity, depression, and lower condom use [82–87]. Integrated prevention services for persons who use drugs are therefore extremely needed and useful for the reduction of HIV in these communities.

### Limitations

In this study, we systematically selected a subset of women who enrolled from 4 US sites for participation based on specific eligibility criteria for the larger observational incidence study. Thus, we acknowledge bias introduced by drawing our sample from a larger cohort determined by sociogeographic risk eligibility criteria – i.e., the study population was not representative of all US communities. Additionally, we limited the amount of transcripts reviewed from the larger sample of qualitative data gathered from this study as we determined these would be sufficient to achieve data saturation for major thematic domains. We therefore recognize that the findings presented herein do not present all thematic facets elicited in analysis. Instead we endeavor to provide a comprehensive overview of the confluence of factors observed in our study that may be influencing women’s HIV risk in the US.

## Conclusions

Findings from this study can inform tailored programming to more effectively address structural, social, and individual risk factors contributing to HIV/AIDS risk among US women [88, 89]. Our study not only helps to align US cities’ prevention activities with the National HIV/AIDS Strategy (NHAS) to identify resources that can be leveraged to reduce HIV incidence among women, but also provides evidence to ensure that a commensurate array of social/peer support services and biomedical interventions and public health investments are directed toward women who most need them in high-risk communities. Such systems-level investigations and implementation approaches help to address HIV prevention in specific jurisdictions pursuant to the NHAS policy, the Enhanced Comprehensive HIV Prevention Planning and Implementation for Metropolitan Statistical Areas Most Affected by HIV/AIDS (ECHPP) demonstration project, and others (e.g., “Local Implementation Efforts”).

Programs emphasizing financial education and resources and those potentially addressing microfinance opportunities in the community may facilitate greater access to prevention education as these efforts have demonstrated success for women in other parts of the world [90–94]. Combined and integrated interventions accounting for multilevel determinants of risk should stress the importance of financial confidence and independence paired with empowerment education on negotiating partner issues such as concurrency, IPV, and building awareness of the role of risk-taking behavior and substance use to ensure greater personal HIV/STI protection [95].

## Abbreviations

ACASI, audio computer-assisted self-interview; HIV/AIDS, human immunodeficiency virus infection/acquired immune deficiency syndrome; HPTN, HIV Prevention Trials Network; ICR, intercoder reliability; IPV, intimate partner violence; PTIDs, participant identification numbers; PTSD, post-traumatic stress disorder; SAVA, substance abuse, IPV, and AIDS; STIs, sexually transmitted infections
